# The association between parents' stress and parental feeding practices and feeding styles: Systematic review and meta‐analysis of observational studies

**DOI:** 10.1111/mcn.13448

**Published:** 2022-10-25

**Authors:** Dina Almaatani, Andreea Zurbau, Farnaz Khoshnevisan, Robert H. J. Bandsma, Tauseef A. Khan, John L. Sievenpiper, Meta Van Den Heuvel

**Affiliations:** ^1^ Department of Nutritional Sciences, Faculty of Medicine University of Toronto Toronto Canada; ^2^ Toronto 3D Knowledge Synthesis and Clinical Trials Unit, Clinical Nutrition and Risk Factor Modification Centre St. Michael's Hospital Toronto Canada; ^3^ Department of Paediatrics University of Toronto Toronto Canada; ^4^ Division of Gastroenterology, Hepatology and Nutrition Hospital for Sick Children Toronto Canada; ^5^ Department of Medicine, Temerty Faculty of Medicine University of Toronto Toronto Ontario Canada; ^6^ Department of Medicine, Division of Endocrinology and Metabolism St. Michael's Hospital Toronto Ontario Canada; ^7^ Li Ka Shing Knowledge Institute St. Michael's Hospital Toronto Ontario Canada; ^8^ Division of Paediatric Medicine Hospital for Sick Children Toronto Canada

**Keywords:** child nutrition, feeding practice, feeding style, general stress, parenting stress, systematic review

## Abstract

In the extended UNICEF framework of early childhood nutrition, parents' stress is associated with parental feeding style. However, no comprehensive review has examined the association between parents' stress and feeding styles and practices. The objective of our review was to synthesise the current literature examining the association between parents' stress and their feeding practices and/or styles, among parents of children ≤ 5 years old. We searched; MEDLINE, EMBASE, PSYCHINFO and CINAHL from 2019 to 2021. Two investigators independently extracted relevant data and assessed the study quality and the certainty of evidence. Data were pooled using generic inverse variance with fixed effects (<5 comparisons) or random effects (≥5 comparisons) and expressed as correlation coefficients with 95% confidence intervals (CI). Between study heterogeneity was assessed using Cochran's Q and quantified with *I* ^2^. We identified 6 longitudinal and 11 cross‐sectional studies, of which 4 studies provided sufficient data to be pooled. A very small correlation between general stress and restrictive feeding practices was observed (*r* = 0.06 [95% CI: 0.01−0.12]; no substantial heterogeneity (*I* ^2^ = 0.00%, *P*_Q_ < 0.85, very low certainty). No correlation between general stress and feeding pressure was identified (*r* = 0.06 [95% CI: −0.02 to 0.15]). Results showed that both general and parenting stress were associated with suboptimal breastfeeding practices and unresponsive feeding styles. Conclusion: This study demonstrated a low‐to‐moderate quality of literature for the inclusion of parents' stress in the extended UNICEF care model of child nutrition. Future research needs to explore this relationship longitudinally and in ethnic diverse populations to inform tailored interventions that promote responsive feeding practices.

## Key messages

- General stress has a small correlation with parental restriction feeding practices.
- Parenting stress is associated with uninvolved feeding styles.
- The specific relation between general and parenting stress and feeding style and/or practice needs to be further explored to inform tailored interventions to promote responsive feeding practices.

## Graphical abstract

This paper provided an extensive review of the literature on the association between parents' stress and their feeding styles. Addressing parents' stress to improve feeding practice and/or style could be an important pathway to improve early childhood nutrition.

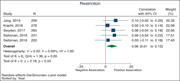

## 1 INTRODUCTION

Parental feeding practices and parental feeding styles are terms that have been used to describe feeding interactions between parents and their children (Shloim et al., 2015). Previous research has found that parental feeding styles and feeding practices are associated with a child's growth and eating behaviours (Gemmill et al., 2013; Shloim et al., 2015; Tovar et al., 2012). Throughout the literature, the terms parental *feeding practices* and *feeding styles* have been used interchangeably, however, they describe different constructs of feeding (Blissett, 2011; Faith et al., 2004; Hughes et al., 2005; Shloim et al., 2015; Ventura & Birch, 2008). Parental feeding practices refer to the strategies and techniques parents may employ to maintain or control the feeding context such as when, what, or how much their children eat (Faith et al., 2004; Thompson et al., 2013; Ventura & Birch, 2008). They are characterised by three main constructs; coercive control (e.g., pressure to eat and restriction); structure/parent's organisation of meal times (e.g., rules and limits, modelling, meal and snack routines) and autonomy support/promoting independence (child involvement, encouragement) (Vaughn et al., 2016). Parental feeding styles can be defined as attitudes or an emotional climate that characterises the parents' approach to their children during feeding interaction (Faith et al., 2004; Ventura & Birch, 2008). Feeding styles are generally classified into four dimensions; authoritative (high demand/high response), authoritarian (high demand/low response), indulgent (low demand/high response) and uninvolved (low demand/low response) (Shloim et al., 2015). As an example, indulgent and uninvolved feeding styles have been associated with a higher intake of low‐nutrient and energy‐dense snacks in children (Hoerr et al., 2009; Hughes et al., 2005). Parental feeding styles are often consistent over time, but parental feeding practices can vary depending on different factors such as a child's weight or temperament (Thompson et al., 2013). Certain feeding practices have been related to the development of inappropriate eating behaviours in children (El‐Behadli et al., 2015). Parents who prompt their children to eat during meals may interfere with the child's internal cues for fullness and hunger which may cause issues with eating self‐regulation (Savage et al., 2007).

Parents' mental health, including stress, has been reported to affect parental feeding style and practices (Bennett et al., 2016; Harwood et al., 1999). *General stress* can be defined as the ability to cope with challenges, or emotional or behavioural responses that an individual may experience toward an unpleasant event (Hackie & Bowles, 2007; Hurley et al., 2008). While *parenting stress* can be conceptualised as psychological distress experienced by parents as a response to their efforts to meet parenting demands (Barroso et al., 2016). In the United States, 13% of children live in households with at least one parent experiencing high parenting stress (Raphael et al., 2010). Elevated levels of stress can influence how parents perceive their child's behaviour, as well as how parents interpret and respond to their child's eating behaviours (Berge et al., 2017; Gemmill et al., 2013; Norman et al., 2015; Powers et al., 2002; Shankardass et al., 2014). Parents' stress was incorporated into the extended UNICEF care model of child nutrition (El‐Behadli et al., 2015). This framework can be used to identify factors that affect nutrition in infancy and early childhood. In this framework parents' stress is associated with parental feeding style (El‐Behadli et al., 2015). As justification, the authors provided a narrative review of eight studies that explored the relationship between maternal mental health and parental feeding style. However, only three of these studies examined parents' stress and feeding practices, with only one study focusing on preschool age (El‐Behadli et al., 2015). Because of the substantial parental influence on feeding practices among young children, it is especially important to investigate the role of parents' stress and feeding practices in early childhood. Furthermore, it is unclear if general stress has a different effect on feeding practices and/or styles than parenting stress; this can be important in the development of interventions to promote healthy nutrition in early childhood. Therefore, the objective of our study was to synthesise the current literature examining parents' stress (both general and parenting stress) and parental feeding practices and style among children ≤5 years old.

## 2 METHODS

This systematic review was conducted according to the Cochrane Handbook for Systematic Reviews of Interventions and the results were reported according to PRISMA and MOOSE guidelines (Hutton et al., 2015). The protocol was registered on ClinicalTrials.gov (registration number: NCT04477941).

### 2.1 Study selection

We searched the following databases: MEDLINE, EMBASE and PSYCHINFO (accessed via Ovid), CINAHL (accessed via Ebsco) from 2019 to 2020, and updated research conducted on April 2021. We included studies with longitudinal and cross‐sectional designs that explored the relationship between parents' stress and feeding practice and/or style among parents of children (≤5 years old). The cut‐off of ≤5 years old was chosen because older children have more feeding interactions outside the home. Because there are different definitions of feeding practices and feeding styles, and both terms are often used interchangeably we included studies that measured *any* of the feeding practices and/or feeding styles described in the introduction. Infant‐feeding practices such as breast milk feeding, formula feeding and supplementary feeding were also included (Karmaus et al., 2017). Articles were identified by combining terms for caregivers (e.g., parent, mother and father), feeding (e.g., feeding behaviour, style, practice, breastfeeding and bottle feeding) and stress (e.g., stress, mental health and maternal health) identified from the literature and discussed with a librarian. The detailed search strategy is described in Supporting Information: Appendix 1, no filters were used. Searches were supplemented with a manual search in the references of the included publications (Supporting Information: Appendix 1). We excluded studies that examined postpartum depression, animal studies and review studies.

### 2.2 Data extraction and quality assessment

Two investigators (D. A. and F. K.) independently reviewed and extracted relevant data and assessed the risk of bias. First, the titles and abstracts of the identified studies were screened for eligibility. Second, full texts were examined in detail based on the eligibility criteria outlined above. Third, references of all the eligible articles were screened for potentially eligible studies that were not captured in the search strategy. Extracted data included cohort characteristics such as ethnicity, education, and household income. Disagreements between the reviewers were resolved by a third investigator (MvdH). For missing data, the authors of the primary publication were contacted. The risk of bias for longitudinal studies were assessed using the Newcastle Ottawa Scale (Wells et al., 2008). The scale awarded 9 points based on cohort selection, ascertainment of the outcomes and comparability. For this review, we selected two comparability variables: studies controlling for parents' age and/or parents' history of mental health. In previous literature, these variables were reported to have a correlation with parents' stress and their feeding practices and/or styles (Russell et al., 2018; Wemakor et al., 2018). The scoring range was classified as a low risk of bias if the total score was between 6 and 9, moderate risk between 4 and 5.5 and high risk for a score between 0 and 3.5 (Wells et al., 2008). For cross‐sectional studies risk of bias was assessed using the National Institutes of Health ‘Quality assessment tool for observational cohort and cross‐sectional studies’ (National Heart & Institute, 2014). The tool included 14 items that assessed potential sources of bias including sample selection, confounding and attrition (National Heart & Institute, 2014). There were three choices for each item: yes, no, and other (cannot determine/not applicable/not reported) (National Heart & Institute, 2014). However, for this review, we excluded 5 items considering that they were not applicable for a cross‐sectional design bringing the total score down to 9 (National Heart & Institute, 2014). The quality rating of the final score was based on the number of listed items answered (YES), with a low risk of bias for a score of 6−9, a moderate risk of bias for a score of 4−5.5 and a high risk of bias for a score of 0−3.5 (National Heart & Institute, 2014).

### 2.3 Meta‐analysis

The outcomes assessed were feeding practices and/or styles. Data was pooled if evidence was available examining the association between a specific type of stress (general or parenting) and a specific feeding practice or style. Correlation between the exposure and outcome needed to be reported, and we prespecified a minimum of two studies. Pearson correlation coefficient and standard errors (SEs) were extracted for each study. If correlations were not provided, they were calculated from beta coefficient and SEs using published formulae (Borenstein et al., 2010).

### 2.4 Data synthesis and analysis

The primary meta‐analysis was conducted in Stata version 16 (StataCorp). Summary estimates were determined by pooling correlations using generic inverse variance with DerSimonian and Laird random effects model (DerSimonian & Laird, 1986). A fixed effects model was used if there were fewer than five studies (Tufanaru et al., 2015). The pooled estimate was expressed as a pooled correlation coefficient with 95% confidence intervals (CIs). Inter‐study heterogeneity was assessed by the Cochrane Q statistic and quantified by the *I* ^2^–statistic, where an *I* ^2^ value ≥ 50% and *P*_Q_ < 0.1 represented evidence of substantial heterogeneity (Higgins JPT et al., 2019).

A sensitivity analysis was performed to investigate sources of heterogeneity by assessing whether any single study influenced summary estimates by the systematic removal of each study with recalculation of the summary estimate. A study was considered influential if it changed the direction or significance of the pooled estimates or the evidence of heterogeneity. If ≥10 study comparisons were available, we further investigated sources of heterogeneity by performing *a priori* sub‐group analyses by parents' sociodemographic characteristics. If ≥10 study comparisons were available, we assessed small‐study effects or publication bias by visual inspection of the funnel plot and using Egger's and Begg's tests (Begg & Mazumdar, 1994; Egger et al., 1997) using log‐transformed extreme contrast risk ratios. If there was evidence of publication bias from significance tests, we used the Duval and Tweedie (2000) trim‐and‐fill method to consider the likely impact of missing studies on the pooled estimate.

### 2.5 Evidence assessment

Two reviewers (D. A. and T. K.) independently assessed the certainty of the evidence using the Grading of Recommendation Assessment, Development and Evaluation (GRADE) system (Guyatt et al., 2008). Observation studies started with low‐quality evidence and can be downgraded or upgraded based on the following criteria: (a) Criteria to downgrade evidence: risk of bias, inconsistency, indirectness, imprecision and publication bias. (b) Criteria to upgrade evidence: large effect size, attenuation by all plausible confounding effects and dose‐response gradient. Disagreements were reconciled by consensus.

## 3 RESULTS

**Figure 1:**
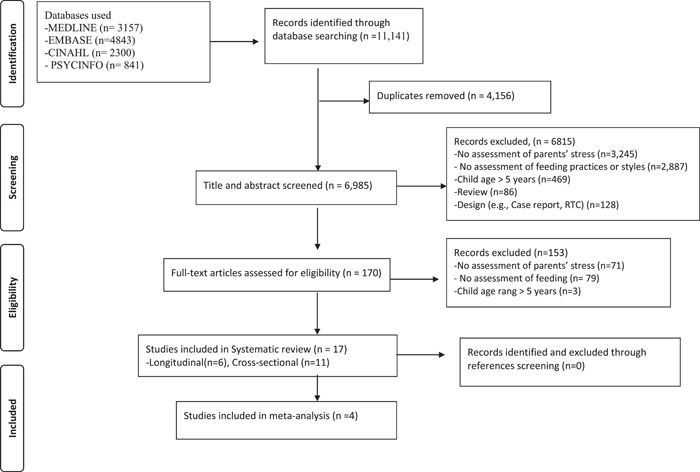
Preferred reporting items for systematic reviews and meta‐analysis flow diagram of systematic review

Figure 1 shows the flow of the literature search and selection. We identified 11,141 papers, of which 17 papers (longitudinal design = 6, cross‐sectional = 11) were included in the final review.

### 3.1 Longitudinal study characteristics

Six longitudinal‐design papers (Table 1) were identified with a sample size ranging from small (*N* = 34) (Park et al., 2016) to relatively large (*N* = 5107) (Webb, 2018). Heterogeneity between the studies was related to differences in their populations; some focused on hospitalised participants who followed a G‐tube weaning programme (Park et al., 2016; Silverman et al., 2013), while others focused on nonhospitalized participants who were followed in an outpatient setting (Swanson et al., 2017; Wambach, 1998) or community participants (Webb, 2018), or a mixture of outpatient and community participants (Ritchie‐Ewing et al., 2019). These studies were all conducted in high‐income settings and included research from the United States (*N* = 4), United Kingdom (*N*= 1) and Australia (*N* = 1).

**Table 1:** Characteristics of the included longitudinal studies

Authors	Country	Design	Sample characteristics	Age (child)	Outcomes	Association	Validated questionaries
General stress and feeding practices/styles							
Webb, (2018)	Australia	National longitudinal study (the infant cohort of the longitudinal study of Australian children)	−*N* = 5107 Community −Mothers ethnicity not reported	8.8 ± 2.6 months	Breastfeeding (Breastfeeding duration)	(+) General stress was predictive of shorter breastfeeding practices.	ITSES/Kessler‐6/BSLEI
Wambach, (1998)	USA	A prospective longitudinal analysis (Pilot study)*	*N* = 41 −Outpatient −Mothers ethnicity Anglo (80%) Mexican American (10%) Other (10%)	Birth—9 weeks postpartum	Breastfeeding Behaviours	(+) A direct association between general stress and breastfeeding was not examined, but Perceived stress was associated with fatigue and in turn was associated with shorter breastfeeding practices	BES/PSS
Swanson et al., (2017)	UK	Longitudinal	*N* = 140 −Outpatient −Mothers ethnicity not reported	Birth to 6−8‐week postpartum	breastfeeding maintenance at 6–8‐weeks postnatal	(−) General stress was not a significant predictor of breastfeeding maintenance	GHQ‐12
Ritchie‐Ewing et al. (2019)	USA	Secondary analysis of longitudinal study	*N* = 70 −Community and outpatient −Mothers ethnicity White or other (52.86%) Black (48.6%)	Birth**—**10 week postpartum	Breastfeeding behaviours (initiation and early cessation rates of breastfeeding)	(−) General stress was not associated with breastfeeding practices	BBS/PSS/NUPDQ
Parenting stress and feeding practices/styles							
Park et al., (2016)	USA	Observational	*N* = 34 Inpatient (NICU) −Mothers ethnicity White (61.3%) Black (35.5%) Hispanic (22.6%) More than one (3.2%)	27.4 ± 2.1 (weeks) based on GA	maternal feeding behaviours	(+) Parenting stress was associated with less developmentally supportive feeding behaviours	CES‐D/CHWS/PSS: NICU/d‐EFS
Ritchie‐Ewing et al. (2019)	USA	Secondary analysis of longitudinal study	*N* = 70 −Community & outpatient −Mothers' ethnicity White or other (52.86%) Black (48.6%)	Birth**—**10 week postpartum	Breastfeeding behaviours (initiation and early cessation rates of breastfeeding)	(+) Pregnancy‐related stress was associated with early breastfeeding cessation	BBS/PSS/NUPDQ
Sliverman et al. (2013)	USA	Retrospective analysis of a prospective and retrospective cohort study	*N* = 77 −Inpatient Mothers (92%) Caucasian (71%) Hispanic (13%) African American (7%) Asian (5%)	4.5 ± 2.2 Years	Feeding practices (Gastrostomy Tube feeding weaning)	(−) Parenting stress was not statistically significant with feeding practices	AYCH/MBQ/PSI‐SF

Abbreviations: AYCH, About Your Child's Health; BBS, Breastfeeding Beliefs Scale, Self‐report of breastfeeding behaviours; BES, Breastfeeding Experience Scale; BSLEI, brief stressful life events inventory; CES‐D, The Centre for Epidemiologic Studies Depression Scale; CHWS, The Child Health Worry Scale; D‐EFS, The maternal feeding actions subscale of the Dynamic‐Early Feeding Skills; GHQ‐12, General Health Questionnaire‐12; ITSES, 2 items from the brief infant and Toddler social and emotional scale; Kessler‐6, stressful life events inventory; MBQ, Mealtime Behaviour Questionnaire; NUPDQ, Revised Prenatal Distress Questionnaire; PSI‐SF, Parenting Stress Index short form; PSS, Perceived Stress Scale; PSS: NICU, A subscale (maternal role alteration subscale) of the Parental Stressor Scale: Neonatal Intensive Care Unit.

#### 3.1.1 General stress and feeding practices and/or styles

Four studies assessed the association between general stress and breastfeeding practices. In two studies, general stress was found to be associated with suboptimal breastfeeding practices (Wambach, 1998; Webb, 2018). In a study by Webb (2018), higher general stress was associated with a shorter duration of breastfeeding. In the study of Wambach (1998), first‐time mothers who reported a high level of general stress also reported a high level of maternal fatigue up to 9 weeks post‐partum, which was associated with breastfeeding difficulties such as insufficient breast milk supply. In two other studies, however, general stress was not significantly associated with breastfeeding practices (Ritchie‐Ewing et al., 2019; Swanson et al., 2017).

#### 3.1.2 Parenting stress and feeding practices and/or styles

Three studies reported on the association between parenting stress and feeding practices. In Park et al. (2016), researchers examined mothers of hospitalised preterm infants and found that levels of parenting stress were associated with less developmentally supportive feeding practices. This study described that higher levels of ‘maternal role stress' interfered with the mother's ability to use feeding strategies such as regulating milk flow and using tactile stimulation (Park et al., 2016). In the study of Ritchie‐Ewing et al. (2019) higher pregnancy‐related stress (which included questions about being worried about taking care of a newborn baby) was associated with early cessation of breastfeeding. In Silverman et al. (2013) study, they examined parenting stress in parents of children with feeding difficulties who were dependent on gastrostomy tube feeding before and after an inpatient behavioural‐based tube weaning protocol. Parenting stress was reduced over time (post‐treatment) and feeding practices (such as ‘I have to force my child to eat’) were significantly improved following the weaning protocol (Silverman et al., 2013).

#### 3.1.3 Quality of the longitudinal studies

The quality of the reviewed studies varied (Supporting Information: Appendix 2), with two studies having a moderate risk of bias, and the remaining studies having a high risk of bias (Wells et al., 2008). Although all papers had a relatively representative sample, they did not demonstrate the baseline feeding practices and/or feeding styles at the beginning of each study. The outcomes were self‐reported in all studies except in the study of (Park et al., 2016) where videotaping was used to assess feeding practices. The follow‐up period for each study was thought to be sufficient for the outcomes to occur, but only three studies reported follow‐up rates (Silverman et al., 2013; Swanson et al., 2017; Wambach, 1998). Only three studies adjusted for the caregivers' age in the analysis (Park et al., 2016; Ritchie‐Ewing et al., 2019; Webb, 2018), and no study accounted for the caregiver's previous history of mental health difficulties. In all six studies, parents' stress was examined only as a variable, not as an independent exposure variable.

### 3.2 Cross‐Sectional studies characteristics

Eleven papers were identified (Table 2) with a cross‐sectional design. The sample size ranged from 265 (Jang et al., 2019) to 840 (Silverman et al., 2021). Studies were conducted in high‐income settings and included the USA (*N* = 7), Iceland (*N* = 1), Australia (*N* = 1), Spain (*N* = 1), and a multi‐country study (*N* = 1). In two studies, participants were recruited from outpatient clinics, (Etowa et al., 2021; Silverman et al., 2021), whereas in the remaining nine studies participants were recruited from the community (Gila‐Díaz et al., 2020; Hughes et al., 2015; Hurley et al., 2008; Jang et al., 2019; Kracht et al., 2018; Rodgers et al., 2014; Saltzman et al., 2016; Swyden et al., 2017; Thome et al., 2006).

**Table 2:** Characteristics of the included cross‐sectional studies

Author	Country	Sample characteristics	Age (Child)	Outcomes	Association (−/+)	Validated questionaries
**General stress and feeding practices/styles**						
Kracht et al. (2018)	USA	−*N*= 278 University campus targeting staff and faculty Mothers' ethnicity Caucasian (73%) African American (5%) Hispanic (6%)	2−5 Year	Feeding Practices [pressure to eat and restriction]	(+) General stress was associated with restrictive feeding practices	CFQ DASS‐21
Swyden et al. (2017)	USA	*N* = 285 Community Mothers' ethnicity not reported	2−5 Year	Feeding practices (Restrictive)	(+) General stress was associated with the use of restrictive feeding practices	DASS‐21/CFQ
Rodgers et al. (2014)	Australia	*N* = 306 Community Mothers' ethnicity not reported	1.5− 2.5 Y	Feeding behaviour (emotional and instrumental child feeding practices)	(+) General stress was associated with emotional feeding practices (i.e., using food to sooth)	DASS‐21/EE‐DEBQ/FR‐CFPQ/EF‐PFSQ/EE‐DEBQ‐PV
Etowa et al. (2021)	Multiple centre (USA/Canada/Africa)	*N* = 690 Outpatient African‐ Caribbean‐ Black mothers with HIV infection	Child not reported only mothers (18−49) years	Determinants of Infant‐feeding practices	(+) General stress was a significant determinant of exclusive formula feeding relative to mixed formula‐ and breastfeeding and exclusive breastfeeding	CPSS/IIFAS
Jang et al. (2019)	USA	*N* = 265 Community, mothers (85.9%) White (53.4%) Hispanic (8.8%) African American (19.1%) Asian (13.9%)	2−5 Year	Feeding Practices (restriction and pressure to eat)	(−) General stress was not statistically significant with pressure to feed and restrictive feeding practices.	PSS/PSS*/FSQ/RP‐CFQ/HFFQ
Saltzman et al. (2016)	USA	*N* = 441 Community, mothers (89.3%) Hispanic (6%) White (71%) African American (19%) Asian (8%)	2−4 Year	Feeding practices (Restrictive)	(−) General stress was not significantly associated with restrictive feeding practices	EDDS/CFPQ/CCNES/DASS‐21
Gila‐Díaz et al. (2020)	Spain	*N* = 711 Community (online) Mothers' ethnicity not reported	Birth‐6 months	Breastfeeding	(−) General stress was not associated with breastfeeding practices	PSS/BAS
Hurley et al. (2008)	USA	*N* = 702 Community, Participants of Special Supplemental Nutrition Programme for Women, Infants and Children (WIC)) Mothers' ethnicity White (49.6%) Hispanic (14.5%) African American (36%)	birth‐13 months	Feeding styles	(+) General stress was significantly associated with forceful and uninvolved feeding styles	FYBS/PSS/PHQ/STAI/IchQ/CFQ/IFQ
Parenting stress and feeding practices/styles						
Thome et al. (2006)	Iceland	*N* = 734 Community Mothers' ethnicity not reported	11.8 weeks ±1.9	Exclusive breastfeeding	(+) Parenting stress was negatively associated with exclusive breastfeeding	EPDS/PSI‐SF/IFQ
Sliverman et al. (2021)	USA	*N* = 840 Outpatient, mothers (92%) Caucasian (75%) Hispanic (8%) African American (7%) Asian (4%)	18 months‐ 5 years	Paediatric feeding disorder (PFD)	(+) Higher level of parenting stress among parents of children with PFD	PSI/MBQ
Jang et al. (2019)	USA	*N* = 265 Community, mothers (85.9%) White (53.4%) Hispanic (8.8%) African American (19.1%) Asian (13.9%)	2−5 Year	Feeding Practices (restriction and pressure to eat)	(−) Parenting stress was not significantly correlated with pressure to feed and restrictive feeding practices	PSS/PSS*/FSQ/RP‐CFQ/HFFQ
Hughes et al. (2015)	USA	*N* = 290 Community, Recruited from Head Start Centres, mothers (96%) African American (45.2%) Hispanic (54.8%)	4.43 ± 0.7	Feeding styles	(+) Parenting stress was associated with uninvolved feeding styles	CFSQ/PSI‐SF/CES‐D

Abbreviations: BAS, Breastfeeding Adherence Score; BFQ, The Behaviour‐Based Feeding Questionnaire; CCNES, Coping with Children's Negative Emotion Scale; CES‐D, Centre for Epidemiologic Studies Depression Scale; CFPQ, Comprehensive Feeding Practices Questionnaire; CFQ, Child Feeding Questionnaire; CFSQ, Caregiver's Feeding Styles Questionnaire; CPSS, Cohen perceived stress scale; DASS‐21, Depression, Anxiety, Stress Scale short form; EDDS, Eating Disorder Diagnostic Scale; EE‐DEBQ, Emotional Eating subscale of the Dutch Eating Behaviour Questionnaire; EE‐DEBQ‐PV = The Emotional Eating subscale of the Dutch Eating Behaviour Questionnaire Parent version; EF‐PFSQ, The five‐item Emotional Feeding subscale from the Parent Feeding Style Questionnaire; EPDS, Edinburgh Postnatal Depression Scale; FR‐CFPQ, Food as a Reward subscale from the Comprehensive Feeding Practices Questionnaire; FSQ, Feeding Strategies Questionnaire; HFFQ, Harvard Service Food Frequency Questionnaire; FYBS, Feeding Your Baby scale; IchQ, fussy‐difficult factor of the Infant Characteristics Questionnaire; IIFAS, Iowa infants feeding attitudes Scales; IFQ, Infant feeding Questionnaire; MBQ, Mealtime Behaviour Questionnaire; PHQ, Primary Care Evaluation of Mental Disorders Patient Health Questionnaire; PSI‐CV, Parenting Stress Index–Chinese Version; PSI‐SF, Parenting Stress Index‐Short Form; PSS, Perceived Stress Scale; PSS*, Parental Stress Scale; RP‐CFQ, Restriction and Pressure to Eat subscales of the Child Feeding Questionnaire; STAI, Spielberger State‐Trait Anxiety Inventor.

#### 3.2.1 General stress and feeding practices and/or styles

Eight studies examined the association between general stress and feeding practices (Etowa et al., 2021; Gila‐Díaz et al., 2020; Hurley et al., 2008; Jang et al., 2019; Kracht et al., 2018; Rodgers et al., 2014; Saltzman et al., 2016; Swyden et al., 2017). Four studies identified an association between general stress and unhealthy feeding practices. In the study of Kracht et al. (2018) there was a significant association between general stress in mothers and restrictive feeding practices but not with pressure to eat. In Swyden et al. (2017) general stress in mothers was also significantly associated with restrictive feeding practices. Rodgers et al. (2014) showed that general stress was positively and significantly correlated with emotional feeding practices (e.g., using food to soothe) but not with instrumental feeding (e.g., using food to reward a child for the desired behaviour). Etowa et al. (2021) showed that general stress was a significant determinant of exclusive formula feeding relative to mixed formula‐ and breastfeeding and relative to exclusive breastfeeding practices. Three papers reported no significant association between general stress and feeding practices (Gila‐Díaz et al., 2020; Jang et al., 2019; Saltzman et al., 2016). Saltzman et al. (2016) explored the effects of parent binge beating and restrictive feeding practices in a community cohort of parents. They also reported the correlation between general stress and two types of restrictive feeding practices ‘restriction for weight control’ and ‘restriction of health’ (Saltzman et al., 2016). General stress was not significantly correlated with any of the restrictive feeding practices in this study (Saltzman et al., 2016). As for the study of Jang et al. (2019) no significant correlation between general stress and pressure to feed practices and/or restrictive feeding practices was identified. Gila‐Díaz et al., 2020 reported that maternal perceived stress was not associated with breastfeeding practices. The study of Hurley et al. (2008) was the only study that examined the association between general stress and feeding style. The results indicated that general stress in low‐income mothers was significantly associated with a forceful and uninvolved feeding style (Hurley et al., 2008).

#### 3.2.2 Parenting stress and feeding practices and/or styles

Four studies examined the association between parenting stress and feeding practices (Howe et al., 2019; Jang et al., 2019; Silverman et al., 2021; Thome et al., 2006). Thome et al. (2006) showed that exclusively breastfeeding mothers had lower mean scores of parenting stress. Silverman et al. (2021) examined the stress levels of caregivers of children with paediatric feeding disorders and demonstrated that children's mealtime behaviours, such as mealtime aggression/distress, were significantly associated with parenting stress. In contrast, Jang et al. (2019) reported no significant correlation between parenting stress, pressure to feed and restrictive feeding practices. The study by Hughes et al. (2015) was the only study that explored parenting stress and feeding style. The authors examined the association between depressive symptoms, parenting stress and parents feeding styles in low‐income families (Hughes et al., 2015). The results showed that parenting stress was higher among parents using uninvolved feeding styles compares to other styles (Hughes et al., 2015).

#### 3.2.3 Quality of the cross‐sectional studies

The reviewed cross‐sectional studies (Supporting Information: Appendix 3) varied between a low risk to moderate risk of bias (National Heart & Institute, 2014). Although all studies reported a research objective and described the study population, not all studies reported the recruitment years of data collection (Jang et al., 2019; Kracht et al., 2018; Rodgers et al., 2014; Swyden et al., 2017). Among the nine studies, only two studies provided a description or justification for the sample size (Jang et al., 2019; Thome et al., 2006). All studies were used for the measurement of feeding practices and styles with reliable and valid questionnaires. Three studies did not adjust for potential confounding variables (Howe et al., 2019; Swyden et al., 2017; Thome et al., 2006).

### 3.3 Meta‐analysis

**Figure 2:**
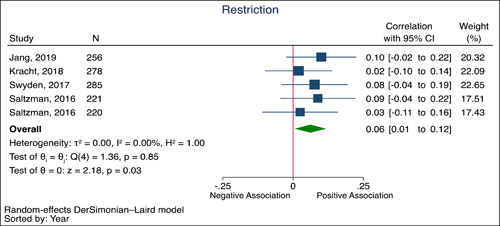
Forrest plot showing the correlation of general stress with feeding restriction Data are expressed as correlations with 95% CIs using the generic inverse variance method modelled by random effects (DerSimonian‐Laird). The independent study correlations are represented by the blue squares with the size of the squares reflecting the weight of the association in the pooled analysis. The pooled correlation estimate is represented by the green diamond. Inter‐study heterogeneity was assessed using the Cochran Q statistic and quantified using the *I* ^2^ statistic, with PQ < 0.10 and *I* ^2^ > 50% considered to be evidence of substantial heterogeneity. *Saltzman et al. (2016), overall sample (*n* = 441) was divided by the two scales used for restriction feeding. CI, confidence interval.

**Figure 3:**
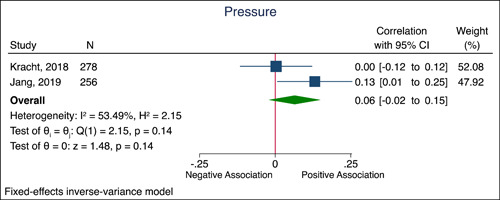
Forrest plot showing the correlation of general stress with feeding pressure Data are expressed as correlations with 95% CIs using the generic inverse variance method modelled by fixed effects. The independent study correlations are represented by the blue squares with the size of the squares reflecting the weight of the association in the pooled analysis. The pooled correlation estimate is represented by the green diamond. Interstudy heterogeneity was assessed using the Cochran Q statistic and quantified using the *I* ^2^ statistic, with PQ < 0.10 and *I* ^2^ > 50% considered to be evidence of substantial heterogeneity. CI, confidence interval.

From the identified papers four studies were eligible for meta‐analysis, these studies examined the correlation between general stress and restrictive feeding practices and the correlation between general stress and feeding pressure practices (Jang et al., 2019; Kracht et al., 2018; Saltzman et al., 2016; Swyden et al., 2017). The results showed a very small correlation between general stress and restrictive feeding practices (5 comparisons; Correlation: 0.06 [95% CI: 0.01−0.12], *P*_correlation_ = 0.03; no substantial heterogeneity, *I* ^2^ = 0.00%, *P*_Q_ < 0.85) (Figure 2). In a pooled analysis there was no evidence of any correlation between general stress and feeding pressure (3 comparisons; Correlation: 0.06 [95% CI: −0.02 to 0.15], *P*_correlation_ = 0.14; no substantial heterogeneity, *I* ^2^ = 53.6%, *P*_Q_ = 0.14) (Figure 3).

The GRADE assessment is summarised in Supporting Information: Table 3. The certainty of the evidence was very low for no association between feeding restriction and general stress owing to a downgrade for serious imprecision. Similarly, the certainty of the evidence was very low for no association between feeding pressure and general stress owing to a downgrade for serious indirectness.

## 4 DISCUSSION

In the extended UNICEF care model of child nutrition, it is suggested that parents' stress is associated with parental feeding style (El‐Behadli et al., 2015). Our study systematically reviewed existing evidence examining associations between parents' stress (both general and parenting stress) and parental feeding practices and style among parents of children younger than five years old. Six longitudinal and eleven cross‐sectional studies were identified. We found low‐to‐moderate quality literature that suggests that parents' stress is related to suboptimal feeding practices and feeding styles in early childhood.

Breastfeeding practices were examined in four longitudinal studies and three cross‐sectional studies. Two studies (Wambach, 1998; Webb, 2018) identified a positive association between general—and parenting stress and early breastfeeding cessation and two studies found a negative association between general stress and exclusive breastfeeding (Etowa et al., 2021; Thome et al., 2006). Three studies did not find any association between general stress or parenting stress and breastfeeding practices (Gila‐Díaz et al., 2020; Ritchie‐Ewing et al., 2019; Swanson et al., 2017). Because of the clinical heterogeneity of the outcomes, it was not possible to conduct a meta‐analysis, therefore no firm conclusion about the relationship between parents' stress and breastfeeding practices could be made. However, it is important to highlight that the largest longitudinal study (*n* = 5107) (Webb, 2018) and a multi‐country cross‐sectional study (*n* = 690) (Etowa et al., 2021) both identified a relationship between self‐reported general stress and shorter breastfeeding duration and exclusive formula feeding respectively, making the relationship between general stress and suboptimal breastfeeding practices likely.

A meta‐analysis that included four cross‐sectional studies showed a very small correlation between general stress and restrictive feeding practices. However, the magnitude of the correlation was very small and the certainty of evidence according to GRADE was very low, due to the quality of the studies included. Two studies examined the association between general stress and pressure to feed in preschool children, a meta‐analysis did not show evidence of any correlation and the certainty of the evidence was also very low (Jang et al., 2019; Kracht et al., 2018). Both restrictive feeding practices and pressure to feed are considered controlling feeding practices. Parents may use restrictive feeding practices to regulate their child's diet or weight (Swyden et al., 2017). Mothers who perceive their children as overweight may restrict their child's food, whereas mothers who perceive their child as underweight may pressure them to eat more food (Tripicchio et al., 2014). One explanation for why we did not find a large correlation between general stress and controlling feeding practices might be that this relationship is moderated by the child's weight and the studies included were conducted in community populations. In other words, in clinical populations of children under or overweight the correlation between general stress and controlling feeding practices might be larger and of clinical importance.

Only two cross‐sectional studies investigated parents' stress and feeding style. General stress was significantly associated with a forceful and uninvolved feeding style (Hurley et al., 2008) and parenting stress was also associated with an uninvolved feeding style (Hughes et al., 2015). Both studies were conducted on low‐income populations, limiting the generalisability of these findings (Hughes et al., 2015; Hurley et al., 2008).

Our systematic review and meta‐analysis have several strengths. We included a systematic search strategy to ensure all published observational studies were identified and pooled. The GRADE approach was used to assess the certainty of evidence. However, our findings need to be interpreted considering some limitations. Many different assessment tools were used to assess parents' stress and feeding practices and this lack of uniformity made it difficult to include studies in the meta‐analysis and influenced the certainty of our results. Because of the limited number of studies, we were not able to explore sources of heterogeneity or make a distinction between the role of general stress and parenting stress and the association between feeding style and/or practice. Many changes in children's eating take place during early childhood (breast milk and/or formula only vs supplementary feeding vs family meals); because of the limited number of studies, no conclusion can be made if meal type influenced the relationship between parental stress and feeding practice. Additionally, in most studies parents' stress was analysed as a variable in the analysis models rather than as an independent variable which made it difficult to interpret specific associations. Importantly, all studies included almost only mothers in their study population, so the results may not be generalisable for fathers and/or other caregivers. Similarly, the ethnicity of the majority of study participants was reported as ‘White’ with only two studies specifically focussing on Black‐African and Hispanic ethnicity (Etowa et al., 2021; Hughes et al., 2015) limiting the generalisability of our results in populations with different ethnicities.

## 5 CONCLUSION

This study provides some evidence for the inclusion of parents' stress in the extended UNICEF care model of early childhood nutrition. Limited low‐to‐moderate quality literature showed that parents' stress is associated with suboptimal breastfeeding practices, restrictive feeding practices and an uninvolved feeding style in early childhood. Addressing parents' stress to improve feeding practice and/or style could be an important pathway to improve early childhood nutrition. Future research should further explore the specific role of general and parenting stress on specific feeding practices and/or styles longitudinally in ethnically diverse populations, as well as different settings. Ultimately, to obtain firm conclusions about the effect of general stress and/or parenting stress on feeding practices, randomised controlled trials are needed that address parents' stress to promote responsive feeding practices in young children.

## AUTHOR CONTRIBUTIONS

Dina Almaatani, Meta Van Den Heuvel and Robert H. J. Bandsma conceptualised and designed the study. Dina Almaatani and Farnaz Khoshnevisan conducted the bibliographic search, collected data, and selected the studies. Dina Almaatani created the main study tables. Meta Van Den Heuvel resolved the disagreements between the first reviewers and reviewed the main study tables. Tauseef A. Khan, Andreea Zurbau carried out the meta‐analysis. Tauseef A. Khan, Andreea Zurbau, Dina Almaatani, Meta Van Den Heuvel and John L. Sievenpiper interpreted the results. Dina Almaatani and Meta Van Den Heuvel drafted the initial manuscript. All authors revised the article critically for important intellectual content. Dina Almaatani and Meta Van Den Heuvel agree to be accountable for all aspects of the work.

## CONFLICTS OF INTEREST

A. Z. is an employee of INQUIS Clinical Research Ltd, a contract research organisation, a consultant for the Glycemic Index Foundation and was funded by a Banting and Best Diabetes Centre Postdoctoral Fellowship.

J. L. S. has received research support from the Canadian Foundation for Innovation, Ontario Research Fund, Province of Ontario Ministry of Research and Innovation and Science, Canadian Institutes of health Research (CIHR), Diabetes Canada, PSI Foundation, Banting and Best Diabetes Centre (BBDC), American Society for Nutrition (ASN), INC International Nut and Dried Fruit Council Foundation, National Honey Board (the US Department of Agriculture [USDA] honey ‘Checkoff’ programme), Institute for the Advancement of Food and Nutrition Sciences (IAFNS; formerly ILSI North America), Pulse Canada, Quaker Oats Centre of Excellence, The United Soybean Board (the USDA soy ‘Checkoff’ programme), The Tate and Lyle Nutritional Research Fund at the University of Toronto, The Glycemic Control and Cardiovascular Disease in Type 2 Diabetes Fund at the University of Toronto (a fund established by the Alberta Pulse Growers), The Plant Protein Fund at the University of Toronto (a fund which has received contributions from IFF), and The Nutrition Trialists Fund at the University of Toronto (a fund established by an inaugural donation from the Calorie Control Council). He has received food donations to support randomised controlled trials from the Almond Board of California, California Walnut Commission, Peanut Institute, Barilla, Unilever/Upfield, Unico/Primo, Loblaw Companies, Quaker, Kellogg Canada, WhiteWave Foods/Danone, Nutrartis, and Dairy Farmers of Canada. He has received travel support, speaker fees and/or honoraria from ASN, Danone, Dairy Farmers of Canada, FoodMinds LLC, International Sweeteners Association, Nestlé, Abbott, General Mills, Comité Européen des Fabricants de Sucre (CEFS), Nutrition Communications, International Food Information Council (IFIC), Calorie Control Council and International Glutamate Technical Committee. He has or has had ad hoc consulting arrangements with Perkins Coie LLP, Tate & Lyle, and Inquis Clinical Research. He is a member of the European Fruit Juice Association Scientific Expert Panel and a former member of the Soy Nutrition Institute (SNI) Scientific Advisory Committee. He is on the Clinical Practice Guidelines Expert Committees of Diabetes Canada, the European Association for the study of Diabetes (EASD), the Canadian Cardiovascular Society (CCS), and Obesity Canada/Canadian Association of Bariatric Physicians and Surgeons. He serves or has served as an unpaid scientific advisor for the Food, Nutrition, and Safety Programme (FNSP) and the Technical Committee on Carbohydrates of IAFNS (formerly ILSI North America). He is a member of the International Carbohydrate Quality Consortium (ICQC), an Executive Board Member of the Diabetes and Nutrition Study Group (DNSG) of the EASD, and a Director of the Toronto 3D Knowledge Synthesis and Clinical Trials foundation. His spouse is an employee of AB InBev. The remaining authors declare no conflicts of interest.

## Supporting information

Supporting information.

## Data Availability

Data available upon request.
