# Supplementary material for: The association between parents' stress and parental feeding practices and feeding styles: Systematic review and meta‐analysis of observational studies
Source: Matern Child Nutr. 2022 Oct 25;19(1):e13448. doi: 10.1111/mcn.13448 (PMC9749598; doi:10.1111/mcn.13448)
Supplement: Supplementary file 1 — Supporting information. [file MCN-19-e13448-s001.docx]

**Table 3:** GRADE for General Stress

|  | **Design**  **Trials (N)** | **Quality assessment** | | | | |  |  | **Effect (MD [95%CI], P_MD_)** | | **Quality** |
| --- | --- | --- | --- | --- | --- | --- | --- | --- | --- | --- | --- |
|  |  | **Downgrades** | | | | |  | **Upgrades** |  |  |  |
|  |  | **Study limitations (Risk of Bias)** | **Inconsistency** | **Indirectness** | **Imprecision** | **Publication bias** |  | **Dose response** |  |  |  |
| **General Stress** | | | | | | | | | | | |
| Feeding restriction | | | | | | | | | | | |
|  | Cross-sectional studies | Not serious | Not serious | Not serious | Serious | NA |  | N/A | ↔ | 0.06 [-0.01, 0.12], P=0.3 | **⨁◯◯◯**  Very low |
| Justification | 5 studies | Majority of studies rated with low/unclear risk of bias | No evidence of significant heterogeneity | Number and type of trial did not limit generalizability of the results | The confidence interval (-0.01 to 0.12) did cross the MID (±0.5 correlation) |  |  |  |  |  |  |
| **Feeding Pressure** | |  |  |  |  |  |  |  |  |  |  |
|  | Cross-sectional studies | Not serious | Not serious | Serious | Not serious | NA |  | N/A | ↔ | 0.06 [0.02, 0.015], P=0.14 | **⨁◯◯◯**  Very low |
| Justification | 2 studies | studies rated with low /unclear risk of bias | No evidence of significant heterogeneity | Only two trials limited generalizability | The confidence interval (-0.02, 0.015) did not cross the MID (±0.5 correlation |  |  |  |  |  |  |

**Appendix 1.** Database and Search Strategy

| **Medline**   1. parents/ or fathers/ or mothers/ 2. Caregivers/ 3. Parenting/ 4. parent-child relations/ or father-child relations/ or mother-child relations/ 5. Maternal Behavior/ 6. Paternal Behavior/ 7. parent*.tw,kf. 8. mother*.tw,kf. 9. father*.tw,kf. 10. (parent* adj3 child* adj3 relat*).tw,kf. 11. (mother* adj3 child* adj3 relat*).tw,kf. 12. ((mother* or matern*) adj3 behavio*).tw,kf. 13. ((mother* or matern*) adj3 practice*).tw,kf. 14. ((mother* or matern*) adj3 style*).tw,kf. 15. caregiver*.tw,kf. 16. (parent* adj3 style*).tw,kf. 17. (parent* adj3 practice*).tw,kf. 18. (parent* adj3 behavio*).tw,kf. 19. 1 or 2 or 3 or 4 or 5 or 6 or 7 or 8 or 9 or 10 or 11 or 12 or 13 or 14 or 15 or 16 or 17 or 18 20. Stress, Psychological/ 21. Maternal Health/ 22. Mental Health/ 23. stress*.tw,kf. 24. (parent* adj3 stress*).tw,kf. 25. (parent* adj3 distress*).tw,kf. 26. ((mother* or matern*) adj3 stress*).tw,kf. 27. ((mother* or matern*) adj3 health*).tw,kf. 28. 20 or 21 or 22 or 23 or 24 or 25 or 26 or 27 29. Feeding Behavior/ 30. Breast Feeding/ 31. Bottle Feeding/ 32. Meals/ 33. (feed* adj3 behavio*).tw,kf. 34. (feed* adj3 style*).tw,kf. 35. (feed* adj3 practice*).tw,kf. 36. (feed* adj3 stress*).tw,kf. 37. (eat* adj3 behavio*).tw,kf. 38. (parent* adj3 feeding*).tw,kf. 39. ((mother* or matern*) adj3 feeding*).tw,kf. 40. (meal* adj3 behavio*).tw,kf. 41. 29 or 30 or 31 or 32 or 33 or 34 or 35 or 36 or 37 or 38 or 39 or 40 42. 19 and 28 and 41 | **PsycINFO**   1. parents/ or fathers/ or mothers/ 2. parenting/ 3. caregivers/ 4. mother child relations/ 5. parent child relations/ 6. parent*.tw. 7. mother*.tw. 8. father*.tw. 9. (parent* adj3 behavio*).tw. 10. (parent* adj3 child* adj3 relat*).tw. 11. (mother* adj3 child* adj3 relat*).tw. 12. ((mother* or matern*) adj3 behavio*).tw. 13. ((mother* or matern*) adj3 practice*).tw. 14. ((mother* or matern*) adj3 style*).tw. 15. caregiver*.tw. 16. (parent* adj3 style*).tw. 17. (parent* adj3 practice*).tw. 18. 1 or 2 or 3 or 4 or 5 or 6 or 7 or 8 or 9 or 10 or 11 or 12 or 13 or 14 or 15 or 16 or 17 19. psychological stress/ 20. mental health/ 21. (parent* adj3 distress*).tw. 22. (parent* adj3 stress*).tw. 23. stress*.tw. 24. ((mother* or matern*) adj3 stress*).tw. 25. ((mother* or matern*) adj3 health*).tw. 26. 19 or 20 or 21 or 22 or 23 or 24 or 25 27. bottle feeding/ 28. breast feeding/ 29. (feed* adj3 behavio*).tw. 30. (feed* adj3 style*).tw. 31. (feed* adj3 practice*).tw. 32. (feed* adj3 stress*).tw. 33. (eat* adj3 behavio*).tw. 34. (parent* adj3 feeding*).tw. 35. ((mother* or matern*) adj3 feeding*).tw. 36. (meal* adj3 behavio*).tw. 37. 27 or 28 or 29 or 30 or 31 or 32 or 33 or 34 or 35 or 36 38. 18 and 26 and 37 |
| --- | --- |
| **Embase Classic+Embase 1**   1. parent/ or father/ or mother/ 2. caregiver/ 3. child parent relation/ or father child relation/ or mother child relation/ 4. maternal behavior/ 5. paternal behavior/ 6. parent*.tw,kw. 7. mother*.tw,kw. 8. father*.tw,kw. 9. (parent* adj3 child* adj3 relat*).tw,kw. 10. (mother* adj3 child* adj3 relat*).tw,kw. 11. ((mother* or matern*) adj3 behavio*).tw,kw. 12. (parent* adj3 behavio*).tw,kw. 13. ((mother* or matern*) adj3 practice*).tw,kw. 14. ((mother* or matern*) adj3 style*).tw,kw. 15. caregiver*.tw,kw. 16. (parent* adj3 style*).tw,kw. 17. (parent* adj3 practice*).tw,kw. 18. 1 or 2 or 3 or 4 or 5 or 6 or 7 or 8 or 9 or 10 or 11 or 12 or 13 or 14 or 15 or 16 or 17 19. mental stress/ 20. maternal welfare/ 21. mental health/ 22. stress*.tw,kw. 23. (parent* adj3 stress*).tw,kw. 24. (parent* adj3 distress*).tw,kw. 25. ((mother* or matern*) adj3 stress*).tw,kw. 26. ((mother* or matern*) adj3 health*).tw,kw. 27. 19 or 20 or 21 or 22 or 23 or 24 or 25 or 26 28. feeding behavior/ 29. breast feeding/ 30. bottle feeding/ 31. meal/ 32. (feed* adj3 behavio*).tw,kw. 33. (feed* adj3 style*).tw,kw. 34. (feed* adj3 practice*).tw,kw. 35. (feed* adj3 stress*).tw,kw. 36. (eat* adj3 behavio*).tw,kw. 37. (parent* adj3 feeding*).tw,kw. 38. ((mother* or matern*) adj3 feeding*).tw,kw. 39. (meal* adj3 behavio*).tw,kw. 40. 28 or 29 or 30 or 31 or 32 or 33 or 34 or 35 or 36 or 37 or 38 or 39 41. 18 and 27 and 40 | CINAHL   1. (MH " Parents") OR (MH "Fathers ") OR (MH " Mothers ") 2. (MH "Caregivers") 3. (MH "Parenting") 4. (MH "Maternal Behavior") 5. (MH "Parental Behavior") OR (MH "Paternal Behavior") 6. (MH "Parent-Child Relations") OR (MH "Father-Child Relations") OR (MH "Mother-Child Relations") OR (MH "Parent-Infant Relations") OR (MH "Father-Infant Relations") OR (MH "Mother-Infant Relations") OR (MH "Parent-Infant Bonding") 7. TI parent* OR AB parent* 8. TI mother* OR AB mother* 9. TI Father* 10. TI ((parent*) N3 (child*) N3 (relat*)) OR AB ((parent*) N3 (child*) N3 (relat*)) 11. TI ((mother*) N3 (child*) N3 (relat*)) OR AB ((mother*) N3 (child*) N3 (relat*)) 12. TI ( ((mother* or matern*) N3 (practice*)) ) OR AB ( ((mother* or matern*) N3 (practice*)) ) 13. TI ( ((mother* or matern*) N3 (style*)) ) OR AB ( ((mother* or matern*) N3 (style*)) ) 14. TI caregiver* OR AB caregiver* 15. TI ((parent*) N3 (style*)) OR AB ((parent*) N3 (style*)) 16. TI ((parent*) N3 (practice*)) OR AB ((parent*) N3 (practice*)) 17. TI ( ((mother* or matern*) N3 (behavio*)) ) OR AB ( ((mother* or matern*) N3 (behavio*)) ) 18. TI ((parent*) N3 (behavio*)) OR AB ((parent*) N3 (behavio*)) 19. 1 OR 2 OR 3 OR 4 OR 5 OR 6 OR 7 OR 8 OR 9 OR 10 OR 11 OR 12 OR 13 OR 14 OR 15 OR 16 OR 17 OR 18 20. (MH "Stress") OR (MH "Stress, Psychological") 21. (MH "Maternal-Child Health") 22. (MH "Mental Health") 23. TI stress* OR AB stress* 24. TI ((parent*) N3 (stress*)) OR AB ((parent*) N3 (stress*)) 25. TI ((parent*) N3 (distress*)) OR AB ((parent*) N3 (distress*)) 26. TI ( ((mother* or matern*) N3 (stress*)) ) OR AB ( ((mother* or matern*) N3 (stress*)) ) 27. TI ( ((mother* or matern*) N3 (health*)) ) OR AB ( ((mother* or matern*) N3 (health*))) 28. 20 OR 21 OR 22 OR 23 OR 24 OR 25 OR 26 OR 27 29. (MH "Eating Behavior") 30. (MH "Breast Feeding") OR (MH "Bottle Feeding") OR (MH "Infant Feeding") 31. (MH "Meals") 32. TI ((feed*) N3 (behavio*)) OR AB ((feed*) N3 (behavio*)) 33. TI feed* N3 style* OR AB feed* N3 style* 34. TI ((feed*) N3 (practice*)) OR AB ((feed*) N3 (practice*)) 35. TI (feed*) N3 (stress*) OR AB (feed*) N3 (stress*) 36. TI (eat*) N3 (behavio*) OR AB (eat*) N3 (behavio*) 37. TI (parent*) N3 (feeding*) OR AB (parent*) N3 (feeding*) 38. TI ( (mother* or matern*) N3 (feeding*) ) OR AB ( (mother* or matern*) N3 (feeding*) ) 39. TI (meal*) N3 (feed*) OR AB (meal*) N3 (feed*) 40. 29 OR 30 OR 31 OR 32 OR 33 OR 34 OR 35 OR 36 OR 37 OR 38 OR 39 41. 19 AND 28 AND 40 |

| **Database** | **Total** |
| --- | --- |
| MEDLINE: April 24^th^, 2021 | 3157 |
| EMBASE: April 24^th^, 2021 | 4843 |
| CINAHL: April 24^th^, 2021 | 2300 |
| PSYCINFO: April 24^th^, 2021 | 841 |
| **Total** | 11.141 |

For all databases, the original search date was 19 November 2019; updated searches were performed on: May 1, 2020; November 30, 2020; April 30, 2021

**Appendix 2:** Quality assessment using Newcastle-Ottawa Assessment Scale for COHORT STUDIES- only one point allotted per category

| Criteria/ Study | Sliverman et al, 2013 | Ritchine-Ewing et al, 2019 | Webb H.J, 2018 | Wambach, K.A., 1998 | Swanson et al, 2017 | Park et al, 2016 |
| --- | --- | --- | --- | --- | --- | --- |
| Selection / 4 | 1 | 1 | 1 | 1 | 2 | 1 |
| Outcomes / 3 | 2 | 1 | 1 | 1 | 2 | 2 |
| Comparability / 2 | 0 | 1 | 1 | 0 | 0 | 1 |
| Total | **3** | **3** | **3** | **2** | **4** | **4** |

The scores rang were classified as low risk of bias if total score were (6-9), moderate risk (4 - 5.5) and high risk for (0- 3.5).

**Appendix 3**: Quality assessment using The National Institutes of Health (NIH) tool for observational cohort and cross-sectional studies

For this tool, the following questions numbers have been removed from final assessments due to not been applicable in cross-sectional studies [ Q 6,7, 10, 12, 13]. That would change the overall scores to (9). The rating: Low risk of bias= 6- 9, moderate risk = 4- 5.5. high risk= 0- 3.5

| Criteria | Kracht, 2018 | Jang, 2019 | Hurley, 2008 | Hughes, 2015 | Swyden, 2017 | Thome, 2006 | Saltzman, 2016 | Rodgers, 2014 | Gila-Diaz et al. 2020 | Etowa et al. 2021 | Sliverman et al. 2021 |
| --- | --- | --- | --- | --- | --- | --- | --- | --- | --- | --- | --- |
| 1. Was the research question or objective in this paper clearly stated? | Yes | Yes | Yes | Yes | Yes | Yes | Yes | Yes | Yes | Yes | Yes |
| 2.Was the study population clearly specified and defined? | Yes | Yes | Yes | No | Yes | Yes | Yes | Yes | Yes | Yes | Yes |
| 3.Was the participation rate of eligible persons at least 50%? | NR | CD | Yes | NR | CD | CD | CD | CD | No | Yes | CD |
| 4.Were all the subjects selected or recruited from the same or similar populations (including the same time period)? Were inclusion and exclusion criteria for being in the study prespecified and applied uniformly to all participants? | CD | CD | Yes | Yes | No | Yes | Yes | Yes | Yes | Yes | Yes |
| 5.Was a sample size justification, power description, or variance and effect estimates provided? | NR | Yes | No | No | No | Yes | No | No | No | Yes | No |
| 8. For exposures that can vary in amount or level, did the study examine different levels of the exposure as related to the outcome (e.g., categories of exposure, or exposure measured as continuous variable)? | Yes | Yes | Yes | Yes | Yes | Yes | Yes | Yes | Yes | Yes | Yes |
| 9. Were the exposure measures (independent variables) clearly defined, valid, reliable, and implemented consistently across all study participants? | Yes | Yes | Yes | Yes | Yes | No | Yes | Yes | Yes | Yes | Yes |
| 11. Were the outcome measures (dependent variables) clearly defined, valid, reliable, and implemented consistently across all study participants? | Yes | Yes | Yes | Yes | Yes | Yes | Yes | Yes | Yes | Yes | Yes |
| 14. Were key potential confounding variables measured and adjusted statistically for their impact on the relationship between exposure(s) and outcome(s)? | Yes | Yes | Yes | Yes | No | No | Yes | Yes | Yes | Yes | Yes |
| TOTAL (YES) | **6** | **7** | **8** | **6** | **5** | **6** | **7** | **7** | **7** | **7** | **9** |
| TOTAL (NO) | **0** | **0** | **1** | **2** | **3** | **2** | **1** | **1** | **2** | **1** |  |
| TOTAL (OTHER) | **3** | **2** | **0** | **1** | **1** | **1** | **1** | **1** |  | **1** |  |
| Quality Rating [Good – Fair – Poor] | **Good** | **Good** | **Good** | **Good** | **Fair** | **Good** | **Good** | **Good** | **Good** | **Good** | **Good** |

*CD = Cannot be determine

*NR= Not reported
